# Prognostic Significance of Heart Failure in Acute Pulmonary Embolism: A Comprehensive Assessment of 30-Day Outcomes

**DOI:** 10.3390/jcm13051284

**Published:** 2024-02-24

**Authors:** Mariam Farid-Zahran, Manuel Méndez-Bailón, José María Pedrajas, Rubén Alonso-Beato, Francisco Galeano-Valle, Vanesa Sendín Martín, Javier Marco-Martínez, Pablo Demelo-Rodríguez

**Affiliations:** 1 Internal Medicine Department, Hospital Universitario Clínico San Carlos, 28040 Madrid, Spain; manmen01@ucm.es (M.M.-B.); jmaria1963@gmail.com (J.M.P.); vanesa.sendin@salud.madrid.org (V.S.M.); javiermarco.z@gmail.com (J.M.-M.); 2 School of Medicine, Universidad Complutense de Madrid, 28040 Madrid, Spain; paco.galeano.valle@gmail.com (F.G.-V.); pbdemelo@hotmail.com (P.D.-R.); 3 Instituto de Investigación Sanitaria Hospital Clínico San Carlos, 28040 Madrid, Spain; 4 Venous Thromboembolism Unit, Internal Medicine Department, Hospital General Universitario Gregorio Marañón, 28007 Madrid, Spain; ralonsob@salud.madrid.org; 5 Instituto de Investigación Sanitaria Gregorio Marañón, 28007 Madrid, Spain; 6 School of Medicine, Universidad CEU San Pablo, 28668 Alcorcón, Spain

**Keywords:** heart failure, venous thromboembolism, pulmonary embolism, mortality, bleeding, comorbidity, left ventricular ejection fraction (LVEF)

## Abstract

Introduction: Patients with heart failure (HF) are known to have an increased risk of pulmonary embolism (PE), but there is limited evidence regarding the prognostic implications of HF in patients with acute PE and the relationship between PE prognosis and left ventricular ejection fraction (LVEF). The primary objective of this study was the development of a composite outcome (mortality, major bleeding, and recurrence) within the first 30 days. The secondary objective was to identify the role of LVEF in predicting the development of early complications in patients with both HF and reduced LVEF. Material and Methods: A prospective study was conducted at two tertiary hospitals between January 2012 and December 2022 to assess differences among patients diagnosed with acute PE based on the presence or absence of a history of HF. Cox regression models were employed to assess the impact of HF and reduced LVEF on the composite outcome at 30 days. Results: Out of 1991 patients with acute symptomatic PE, 7.13% had a history of HF. Patients with HF were older and had more comorbidities. The HF group exhibited higher mortality (11.27% vs. 4.33%, *p* < 0.001) and a higher incidence of major bleeding (9.86% vs. 4.54%, *p* = 0.005). In the multivariate analysis, HF was an independent risk factor for the development of the composite outcome (HR 1.93; 95% CI 1.35–2.76). Reduced LVEF was independently associated with a higher risk of major bleeding (HR 3.44; 95% CI 1.34–8.81). Conclusion: In patients with acute pulmonary embolism, heart failure is independently associated with a higher risk of early complications. Additionally, heart failure with reduced LVEF is an independent risk factor for major bleeding.

## 1. Introduction

Venous thromboembolism (VTE), which includes deep vein thrombosis (DVT) and pulmonary embolism (PE), is a multifactorial disease often associated with other comorbidities. PE ranks as the third-most-common cardiovascular disease, following acute myocardial infarction (AMI) and stroke [1]. The annual incidence rate of VTE is 1–2 cases per 1000 individuals in the general population and increases exponentially with age, reaching 1 case per 100 individuals over the age of 80 years [1,2]. Incidence has begun to show a growing trend in recent years [3], likely due to the aging of the population and the higher prevalence of comorbidities associated with VTE itself, such as obesity, heart failure (HF), and cancer, as well as immobility associated with surgery or hospitalization for medical illness [4]. 

Previous studies have reported that HF is an independent risk factor for the development of VTE [5,6], largely due to the prothrombotic state observed in these patients [7,8]. It is estimated that chronic HF may be present in the medical history of up to 10–20% of patients who develop a thrombotic event [9]. Furthermore, various studies have demonstrated that a thrombotic event during a hospitalization for acute HF is associated with increased morbidity, mortality, and risk of readmission [10,11,12].

However, there are few studies that analyze the implications of a history of HF in patients with acute PE in terms of both its presentation and its short- and long-term outcomes. Various prognostic tools have been developed to analyze the risk of recurrence, mortality, and bleeding in patients who develop a thrombotic event, and few include HF among their parameters [13,14,15]. The Pulmonary Embolism Severity Index (PESI) [15] and its simplified version (PESIs) [16] do include HF as a risk factor for early mortality in patients with PE. However, other studies have been unable to establish HF as an independent prognostic factor in patients with PE [17]. Regarding the risk of bleeding, there is no clear consensus on the impact that a history of HF may have on patients with acute PE. While some authors argue that HF increases the risk of major bleeding in these patients [18,19], others have not found a clear association [20]. 

There is a growing recognition of prognostic differences among patients with HF based on the left ventricular ejection fraction (LVEF), suggesting that patients with HF and reduced LVEF should be considered as a subgroup with a poorer prognosis [21]. However, none of the scores currently used for prognosis in VTE reflect the prognostic difference between patients with HF who have reduced LVEF and those who have preserved LVEF, even though this consideration is assessed in other cardiovascular diseases with high prevalence and morbidity–mortality [22].

The objective of this study is to assess the risk of developing early complications (within the first 30 days) in patients with a history of HF who develop acute PE. The secondary objective is to evaluate the role of LVEF in the early prognosis of patients with HF who develop acute PE.

## 2. Method

### 2.1. Study Design

An observational and prospective study was conducted at two tertiary hospitals in the same region to assess differences among patients diagnosed with symptomatic acute PE based on the presence or absence of a history of HF. Baseline characteristics, presentation patterns, and outcomes (mortality, major bleeding, and recurrence) were compared between groups. The study included a sub-analysis that evaluated differences among patients with and without reduced LVEF.

### 2.2. Study Population

Consecutive patients aged 18 or older who were diagnosed with symptomatic acute PE between January 2012 and December 2022 and who had a follow-up period of at least 30 days were included. Patients with an incidental PE diagnosis or those with a follow-up of less than 30 days were excluded. The diagnosis of PE was confirmed through CT pulmonary angiography or lung scintigraphy. Patients were classified as having HF if they had a documented diagnosis of HF prior to the episode of PE. Patients with HF were further subclassified based on LVEF into those with preserved LVEF (LVEF ≥ 50%) and those with reduced LVEF (LVEF < 49%).

### 2.3. Data Collection

Data regarding the baseline characteristics and risk factors for the development of VTE were collected. Additionally, information about clinical presentation, laboratory and imaging tests, and anticoagulant treatment was also gathered.

### 2.4. Outcomes

The primary objective of the study was to assess rates of development of a composite event that includes all-cause mortality, major bleeding, and recurrence during the first 30 days following the diagnosis of PE in patients with a history of HF. The secondary objective was to assess rates of development of the composite event in the subgroup of patients with HF and reduced LVEF. Major bleeding was defined according to ISTH guidelines as fatal bleeding and/or symptomatic bleeding in a critical area or organ, such as intracranial, intraspinal, intraocular, retroperitoneal, intra-articular, or pericardial bleeding; intramuscular bleeding with compartment syndrome; and/or bleeding causing a fall in hemoglobin levels ≥1.24 mmol/L (20 g/L) or bleeding leading to a transfusion of two or more units of whole blood or red blood cells [23]. PE recurrence was defined as a new intraluminal filling defect on chest CT or a new ventilation–perfusion mismatch on lung scan.

### 2.5. Statistical Analysis

Patient characteristics were presented as frequencies and percentages for qualitative variables. Quantitative variables were expressed as mean ± standard deviation (SD) or median with interquartile range (IQR), depending on the normality and homogeneity of the sample, as assessed using a Shapiro-Francia test and Levene’s test for variance homogeneity, respectively. The association between qualitative variables was evaluated using a chi-square test and Fisher’s exact test. For numerical variables, a *t*-test or Mann-Whitney U test was used, depending on the normality of the variable.

Unadjusted and adjusted Cox regression models were used. The models were adjusted for age, sex, chronic kidney disease (CKD), cancer, thrombocytopenia (platelets < 50,000), recent bleeding (in the last month), and anemia (hemoglobin < 13 g/dL in males and <12 g/dL in females), which were considered the most important confounders. These models were employed to determine the effect of both overall HF and HF with reduced LVEF on risk of the composite event at 30 days (mortality, major bleeding, and recurrence), as well as the effects of these factors on the separate risks of mortality, major bleeding, and recurrence at 30 days. The Kaplan-Meier method was used for graphical representation. A *p*-value < 0.05 was considered significant for all statistical tests. IBM SPSS Statistics for Windows, Version 21.0 (IBM Corp., Armonk, NY, USA), was used for all calculations.

## 3. Results

### 3.1. Baseline Characteristics and Presentation of PE Event

A total of 1991 patients with a diagnosis of symptomatic acute PE, 142 (7.13%) of whom had a history of HF, were included. Baseline characteristics and risk factors for VTE are summarized in Table 1. Patients with HF were significantly older (82.5 vs. 68 years) and had a higher rate of history of ischemic heart disease (20.42% vs. 4.67%), cerebrovascular disease (19.72% vs. 5.21%), peripheral artery disease (9.86% vs. 2.39%), diabetes (28.87% vs. 14.75%), hypertension (88.02% vs. 47.24%), and atrial fibrillation (18.28% vs. 2.02%). Regarding provoking factors for VTE, patients with HF more frequently had undergone immobilization before the event (39.44% vs. 28.18%), with no differences in the other factors.

Patients without a history of HF presented at the time of the event with a higher frequency of chest pain (39.97% vs. 26.76%) and tachycardia (32.02% vs. 23.94%) and a higher thrombotic burden defined by a higher frequency of PE in main arteries (35.05% vs. 23.94%) and concomitant DVT (26.66% vs. 18.31%). Patients in the HF group had more renal insufficiency (50.70% vs. 21.25%), elevated troponin levels (60.55% vs. 40.78%), and elevated natriuretic peptide levels (77.48% vs. 44.63%). Information regarding the presentation and treatment received is available in Table 2.

### 3.2. Outcomes and Follow-Up

**Figure 1:**
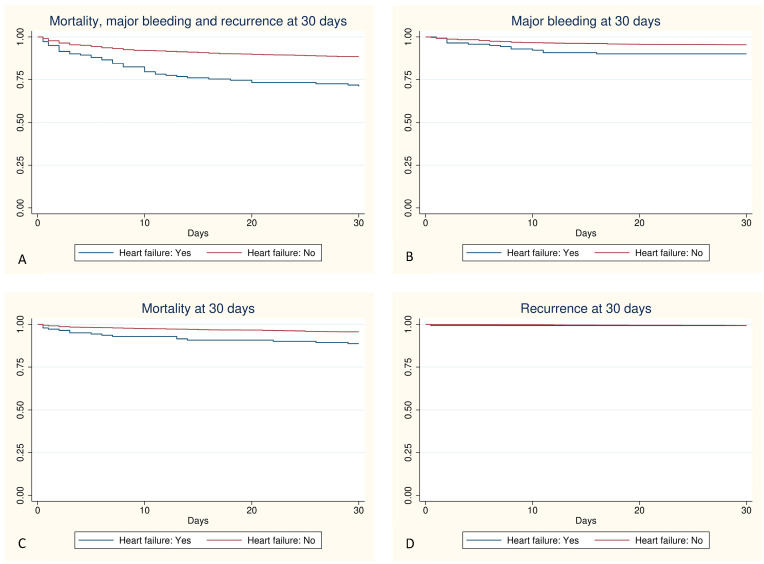
Graphic representation using the Kaplan-Meier method of the outcomes at 30 days; (**A**): composite event, *p* < 0.001; (**B**): major bleeding, *p* = 0.004; (**C**): mortality, *p* = 0.001; (**D**): recurrence, *p* = 0.934.

Clinical outcomes at 30 days are summarized in Table 3. A total of 96 patients died during the 30-day follow-up, with higher mortality in the HF group (11.27% vs. 4.33%, *p* < 0.001). Both total bleeding and major bleeding were more frequent in the HF group (19.01% vs. 8.06%, *p* < 0.001; and 9.86% vs. 4.54%, *p* = 0.005, respectively). No significant differences were observed in VTE recurrence between the groups (Figure 1).

In the multivariate analysis adjusted for age, sex, chronic kidney disease, cancer, thrombocytopenia, recent bleeding, and anemia, a higher frequency of the composite event was observed in the HF group (HR 1.93; 95% CI 1.35–2.76). In the bivariate analysis, the HF group showed higher rates of mortality (HR 2.70; 95% CI 1.58–4.63) and major bleeding (HR 2.22; 95% CI 1.26–3.92), but these differences were not present in the multivariate analysis. In the sub-analysis of patients with HF and reduced LVEF (*n* = 24, 20.87% of patients with HF), these patients presented a higher risk of major bleeding in the multivariate analysis (HR 3.44; 95% CI 1.34–8.81), but HF and reduced LVEF was not independently associated with mortality or VTE recurrence (Table 4).

## 4. Discussion

In the present study, patients with symptomatic acute pulmonary embolism and a history of heart failure had almost twice the risk of early complications in the first 30 days compared to those without a history of heart failure. This risk was independent of age, sex, or the presence of comorbidities such as renal disease, cancer, thrombocytopenia, recent bleeding, or anemia. Additionally, reduced LVEF was found to be an independent risk factor for the development of major bleeding events in the first 30 days after acute pulmonary embolism. This finding highlights the need to acknowledge HF and reduced LVEF as potential complicating factors in the management of acute PE. Clinicians should closely monitor these patients and consider tailored early-management strategies and follow-up care protocols for better outcomes. 

Previous studies have indicated that a history of HF is a risk factor for developing both in-hospital and long-term complications among patients with PE. Death is one of the most frequently described complications; however, the literature is not unanimous in considering heart failure as an independent risk factor [17,24]. In the bivariate analysis in our study, we observed higher mortality in the HF group (HR 2.70; 95% CI 1.58–4.63), but this difference was not present in the multivariate analysis, suggesting that other comorbidities may contribute to the observed mortality risk. It is described in the literature [9,18,19,24,25,26] that patients with chronic heart failure who present with acute PE are often older and more frequently have other comorbidities such as chronic obstructive pulmonary disease (COPD), anemia, or ischemic heart disease, which we also observed in our study. However, patients with HF have poorer pulmonary and cardiac reserve, resulting in lower tolerance to the acute PE episode and an inability to cope with its hemodynamic and ventilatory demands, potentially exacerbating outcomes in this population [18,19,24,26]. Additionally, in our study, we observed that patients with HF had a higher frequency of hypoxemia at the time of acute PE, a finding also described in the literature [18]. In an analysis of clinical predictors of fatal pulmonary embolism, Laporte et al. [17] observed a two-to-three times higher risk in patients with heart disease—including HF—although they could not confirm HF as an independent risk factor in their validation model. Another comparative study of patients with acute PE, conducted by Monreal et al. [24], found higher crude mortality in patients with COPD (12%) and HF (17%), although they did not perform a multivariate analysis. In contrast, Piazza et al. [19] did observe that HF was independently associated with higher mortality, both during hospitalization (OR 2.04; 95% CI 1.15–3.62) and at 30 days of follow-up (OR 1.57; 95% CI 1.01–2.43).

Regarding the risk of bleeding, there is no consensus regarding the role that HF might play in patients presenting with acute PE. While some studies describe a history of HF as a risk factor for major bleeding [18,19], others have not found such an association [20]. Additionally, several prediction models have been developed to assess the individual risk of major bleeding during the first three months of anticoagulant therapy initiation in patients with VTE [13,27,28,29,30,31,32,33]; in these models, chronic HF was assessed as a risk factor for bleeding, but none of the associated studies described heart failure as an independent risk factor. Ducrocq et al. [34] demonstrated that beyond associated comorbidities, HF itself is a risk factor for major bleeding. In previous research, a history of HF has been identified as a risk factor for bleeding in patients presenting with a myocardial infarction [35,36], and acute HF is a component of the CRUSADE score, which is used to predict bleeding in these patients [37]. In the bivariate analysis used in our study, we observed an association between heart failure and a higher risk of major bleeding (HR 2.22; 95% CI 1.26–3.92), an observation that did not persist in the multivariate analysis. However, we found that LVEF was an independent risk factor for major bleeding (HR 3.44; 95% CI 1.34–8.81). In this regard, the increased cardiac stress, reduced cardiac output and frailty described in patients with reduced LVEF could contribute to the observed increase in the risk of bleeding [38,39]. Regarding these findings, strategies to reduce the risk of bleeding in these patients should be considered, and reduced LVEF should possibly be considered as a distinct risk category. Individualized approaches to anticoagulant therapy may improve outcomes and reduce complications in this population. In these fragile patients, tailored anticoagulant treatment, including the choice of drugs with reduced bleeding risk and fewer pharmacological interactions, should be considered; this step can ensure that these patients are offered the safest and most suitable options for their situation [40]. 

In exploring prognostic differences among patients with HF, increasing attention has been given to LVEF as a distinguishing factor, and several studies suggest considering patients with reduced LVEF as a subgroup with a worse prognosis [21,41]. In the context of PE, some authors claim that reduced LVEF has independent predictive value for in-hospital mortality, which is not the case for patients with preserved LVEF [9,25,42]. Additionally, reduced myocardial contractility and the use of beta-blockers in patients with reduced LVEF can worsen their inotropic and chronotropic responses to PE. There is also a significantly reduced capacity to compensate for hypoxemia during PE, which may thus have an even more detrimental effect on patients with reduced LVEF, causing myocardial ischemia. However, Ovradovic et al. [9] did not observe a significant association between any heart-failure phenotype and 30-day mortality. Similarly, in our study, we did not observe differences in mortality based on LVEF. Regarding bleeding risk in patients with acute PE and reduced LVEF, we are not aware of any studies in the literature that analyze this association. The results of our research support the importance of differentiating between patients with a history of heart failure with reduced LVEF and similar patients with preserved LVEF, as bleeding risk at 30 days is higher in the former case.

Among the limitations of the study, the observational design means that treatment decisions were made by physicians for each patient. Furthermore, conducting this study at two tertiary-level hospitals may limit the representativeness of our study population, as it may not fully capture the diversity of patients seen in other healthcare settings. Additionally, the limited number of patients in the subgroup of patients with reduced LVEF could affect the ability of the study to obtain significant differences in the incidence of some of the events. Among the strengths of this study, this is the first study that provides a comprehensive assessment of the risk of early complications in patients with a history of HF following symptomatic acute PE. The combined perspective on these risks provides a more complete understanding of the clinical implications in this specific population and underscores the necessity of recognizing HF and reduced LVEF as potential complicating factors in the management of acute PE; these findings suggest that clinicians should adopt tailored early-management strategies and follow-up-care protocols for improved outcomes [43,44]. The results significantly reinforce the prognostic importance of HF in relation to early complications after symptomatic acute PE and contribute to the clinical understanding of the evolution of patients with this combination of medical conditions. Furthermore, this study reveals that reduced LVEF is an independent risk factor for bleeding events in this context. This discovery provides a new perspective on the relationship between cardiac function and bleeding events, filling a gap in the existing literature. Future investigations should focus on elucidating the mechanisms underlying the observed differences in outcomes, refining risk-stratification strategies, and identifying optimal treatment approaches for this vulnerable patient population. 

## 5. Conclusions

In conclusion, our study provides compelling evidence that heart failure is independently linked to an elevated risk of early complications following symptomatic acute pulmonary embolism. Furthermore, the identification of reduced LVEF as an independent risk factor for major bleeding emphasizes the need for particular considerations in managing this specific subgroup of patients.

## Figures and Tables

**Table 1:** Baseline characteristics and risk factors for VTE.

Variables	HF (*n* = 142)	Non-HF (*n* = 1849)	*p*-Value
Age (median)	82.5 (IQR 76–88)	68 (IQR 54–79)	<0.001
Sex (male)	62 (43.66%)	892 (48.40%)	0.276
Previous conditions			
Ischemic heart disease	29 (20.42%)	86 (4.67%)	<0.001
Cerebrovascular disease	28 (19.72%)	96 (5.21%)	<0.001
Peripheral artery disease	14 (9.86%)	44 (2.39%)	<0.001
Smoking	10 (7.09%)	243 (13.27%)	0.034
Diabetes	41 (28.87%)	272 (14.75%)	<0.001
Arterial hypertension	125 (88.02%)	872 (47.24%)	<0.001
Atrial fibrillation	25 (18.28%)	36 (2.02%)	<0.001
Dyslipidemia	80 (56.34%)	558 (30.31%)	<0.001
COPD	36 (25.35%)	203 (10.98%)	<0.001
Obstructive sleep apnea	12 (8.45%)	85 (4.60%)	0.04
Cirrhosis	1 (0.70%)	15 (0.81%)	1
Chronic kidney disease	50 (35.21%)	177 (9.57%)	<0.001
Bleeding in the last month	5 (3.52%)	71 (3.84%)	0.848
Risk factors for VTE			
Previous PE or DVT	11 (7.75%)	168 (9.09%)	0.591
Family history of VTE	3 (3.53%)	83 (6.65%)	0.361
Unprovoked VTE	59 (41.55%)	814 (44.02%)	0.567
Provoked VTE	83 (58.45%)	1035 (55.98%)	0.567
Cancer	30 (21.13%)	318 (17.20%)	0.235
Recent immobilization	56 (39.44%)	521 (28.18%)	0.004
Recent surgery	12 (8.45%)	210 (11.36%)	0.289
Long travel	0 (0%)	40 (2.18%)	0.11
Hormone therapy	8 (5.67%)	141 (7.10%)	0.521

HF: heart failure; IQR: interquartile range; COPD: chronic obstructive pulmonary disease; VTE: venous thromboembolism; PE: pulmonary embolism; DVT: deep vein thrombosis.

**Table 2:** Clinical presentation and treatment received.

Variables	HF (*n* = 142)	Non-HF (*n* = 1849)	*p*-Value
Clinical presentation			
Heart rate > 100 bpm	34 (23.94%)	592 (32.02%)	0.046
SBP <90 mmHg	12 (8.45%)	99 (5.35%)	0.121
Respiratory rate >20 rpm	14 (41.18%)	131 (26.84%)	0.071
O_2_ saturation <90%	23 (33.33%)	152 (22.22%)	0.037
Required hospital admission	116 (95.87%)	1443 (96.01%)	0.812
Concomitant DVT	26 (18.31%)	493 (26.66%)	0.029
Dyspnea	117 (82.39%)	1438 (80.21%)	0.723
Syncope	26 (18.32%)	249 (13.47%)	0.223
Chest pain	38 (26.76%)	739 (39.97%)	0.006
Hemoptysis	6 (4.23%)	52 (2.81%)	0.481
Image tests			
PE in main arteries	34 (23.94%)	648 (35.05%)	0.007
PSAP (median)	43 (IQR 25–55)	37 (IQR 29–49)	0.001
Right atrium dilatation	27 (19.01%)	138 (7.46%)	<0.001
Right ventricle hypokinesia	40 (32.79%)	489 (34.66%)	0.677
TAPSE <17 mm	14 (21.21%)	150 (16.84%)	0.363
Preserved LVEF (≥50%)	91 (79.13%)	NA	NA
Reduced LVEF (<49%)	24 (20.87%)	NA	NA
Laboratory tests			
Hemoglobin <12 g/dL in women	28 (35%)	276 (28.93%)	0.253
Hemoglobin <13 g/dL in men	31 (50%)	233 (26.03%)	<0.001
Platelets <100,000	8 (5.63%)	44 (2.38%)	0.028
D-Dimer (ng/mL) (median)	2889 (IQR 1176–5426)	2716.5 (IQR 1150–6355.5)	0.484
Renal insufficiency	72 (50.70%)	391 (21.25%)	<0.001
Elevated troponin	66 (60.55%)	551 (40.78%)	<0.001
Elevated NT-ProBNP	86 (77.48%)	528 (44.63%)	<0.001
Acute treatment			
Inferior vena cava filter	6 (4.23%)	76 (4.11%)	0.947
LMWH	129 (90.85%)	1734 (93.78%)	0.169
UFH	14 (9.86%)	259 (14.01%)	0.166
Fibrinolytics	7 (4.93%)	130 (7.03%)	0.34
Vitamin K antagonists	2 (1.41%)	10 (0.54%)	0.209
DOAC	5 (3.52%)	217 (11.74%)	0.003

HF: heart failure, SBP: systolic blood pressure; DVT: deep vein thrombosis; PE: pulmonary embolism; PSAP: pulmonary arterial systolic pressure; TAPSE: tricuspid annular plane systolic excursion; LVEF: left ventricular ejection fraction; NT-proBNP: N-terminal pro-B-type natriuretic peptide, LMWH: low-molecular-weight heparin; UFH: unfractionated heparin; DOAC: direct oral anticoagulants; IQR: interquartile range.

**Table 3:** 30-day clinical outcomes.

Variables	HF (*n* = 142)	Non-HF (*n* = 1849)	*p*-Value
Hospital stay (median, days)	8 (IQR 6–13)	8 (IQR 5–11)	0.076
Overall mortality	16 (11.27%)	80 (4.33%)	<0.001
Bleeding	0 (0%)	7 (8.75%)	0.597
Pulmonary embolism	3 (18.75%)	25 (31.25%)	0.382
Other	13 (81.25%)	48 (60%)	0.024
VTE recurrence	1 (0.70%)	12 (0.65%)	1
Pulmonary embolism	1 (100%)	5 (41.67%)	0.462
Isolated DVT	0 (0%)	5 (41.67%)	1
Other	0 (0%)	2 (16.66%)	1
Major bleeding	14 (9.86%)	84 (4.54%)	0.005
Total bleeding	27 (19.01%)	149 (8.06%)	<0.001

HF: heart failure; VTE: venous thromboembolism; DVT: deep vein thrombosis; IQR: interquartile range.

**Table 4:** Bivariate and multivariate analysis at 30-day follow-up for patients with heart failure and for the subgroup of patients with heart failure and reduced left ventricular ejection fraction (LVEF).

Variables	Unadjusted HR	Adjusted HR **
HF (all)		
Compound event *	2.75 (95% CI 1.96–3.84)	1.93 (95% CI 1.35–2.76)
Major bleeding	2.22 (95% CI 1.26–3.92)	1.59 (95% CI 0.87–2.91)
Death	2.70 (95% CI 1.58–4.63)	1.48 (95% CI 0.84–2.61)
Recurrence	1.08 (95% CI 0.14–8.37)	1.66 (95% CI 0.20–13.59)
HF with reduced LVEF		
Compound event *	3.06 (95% CI 1.51–6.19)	2.05 (95% CI 0.99–4.21)
Major bleeding	4.68 (95% CI 1.90–11.51)	3.44 (95% CI 1.34–8.81)
Death	1.91 (95% CI 0.47–7.78)	1.08 (95% CI 0.26–4.44)
Recurrence	NC	NC

* major bleeding, death or recurrence. ** adjusted for: age, sex, chronic kidney disease, cancer, thrombocytopenia, recent bleeding and anemia. HR: hazard ratio; HF: heart failure; LVEF: left ventricular ejection fraction; NC: not calculated.

## Data Availability

The data presented in this study are available on request from the corresponding author.
